# Small molecules for cell reprogramming: a systems biology analysis

**DOI:** 10.18632/aging.203791

**Published:** 2021-12-17

**Authors:** Anna Knyazer, Gabriela Bunu, Dmitri Toren, Teodora Bucaciuc Mracica, Yael Segev, Marina Wolfson, Khachik K. Muradian, Robi Tacutu, Vadim E. Fraifeld

**Affiliations:** 1 The Shraga Segal Department of Microbiology, Immunology and Genetics, Center for Multidisciplinary Research on Aging, Ben-Gurion University of the Negev, Beer-Sheva, Israel; 2 Systems Biology of Aging Group, Institute of Biochemistry of the Romanian Academy, Bucharest, Romania; 3 D.F. Chebotarev Institute of Gerontology of National Academy of Medical Sciences of Ukraine, Kiev, Ukraine

**Keywords:** cell reprogramming, chemically-induced pluripotency, chemical-protein interactions, protein-protein interaction networks, longevity pathways

## Abstract

If somatic stem cells would be able to maintain their regenerative capacity over time, this might, to a great extent, resolve rejuvenation issues. Unfortunately, the pool of somatic stem cells is limited, and they undergo cell aging with a consequent loss of functionality. During the last decade, low molecular weight compounds that are able to induce or enhance cell reprogramming have been reported. They were named “Small Molecules” (SMs) and might present definite advantages compared to the exogenous introduction of stemness-related transcription factors (e.g. Yamanaka’s factors). Here, we undertook a systemic analysis of SMs and their potential gene targets. Data mining and curation lead to the identification of 92 SMs. The SM targets fall into three major functional categories: epigenetics, cell signaling, and metabolic “switchers”. All these categories appear to be required in each SM cocktail to induce cell reprogramming. Remarkably, many enriched pathways of SM targets are related to aging, longevity, and age-related diseases, thus connecting them with cell reprogramming. The network analysis indicates that SM targets are highly interconnected and form protein-protein networks of a scale-free topology. The extremely high contribution of hubs to network connectivity suggests that (i) cell reprogramming may require SM targets to act cooperatively, and (ii) their network organization might ensure robustness by resistance to random failures. All in all, further investigation of SMs and their relationship with longevity regulators will be helpful for developing optimal SM cocktails for cell reprogramming with a perspective for rejuvenation and life span extension.

## INTRODUCTION

The pool of adult stem cells is limited, and they undergo cell aging with a consequent loss of functionality [1–3]. This limits the application of adult stem cells for cell replacement therapy. Induced pluripotency (iP), a state where somatic differentiated cells become functionally similar to embryonic stem cells (ESC), may serve as an alternative solution. The breakthrough findings of iP, first discovered by Takahashi and Yamanaka in 2006, by ectopic overexpression of four stemness-related transcription factors (TFs: Oct3/4, Sox2, Klf4, and c-Myc; OSKM in short), in mouse fibroblasts [4], and then repeated in human fibroblasts [5], proved the plasticity potential of differentiated cells to rejuvenate back to the ESC-like state. Since then, various combinations of transcription factors for iP have been proposed [6–8]. Still, the exogenous introduction of transgenes provides a low yield, both *in vitro* and *in vivo*, and may have undesirable complications, including tumorigenicity (reviewed by [3]).

Recently, a number of small molecules (SMs) that are able to induce or enhance pluripotency have been discovered [9–11]. They have definite advantages and could be used for iP as a much safer alternative [12]. First of all, cell dedifferentiation activity could be fine-tuned by varying the concentrations of SM. When needed, the application of lineage-alternating SMs could induce cell differentiation and inhibit cell proliferation. Moreover, SMs are distinguished by non-immunogenicity, cost-efficiency, minimal residual effects on the genome, and feasibility of *in vivo* application [13, 14]. Consequently, this strategy may have great potential in clinical practice. With this in mind, the major goal of this study was to provide a systems biology view of the SMs, thus supporting researchers with a potential basis for the optimal selection of drugs for cell reprogramming.

In this *in silico* study we performed: (i) a comprehensive data mining of SMs; (ii) the characterization of SMs and SM cocktails, including assessing their protein targets and possible interactions between them; (iii) the analysis of pathways targeted by SMs, (iv) the comparison of targets and pathways of SM cocktails with those of the OSKM TFs, and (v) screening for SMs as human metabolites.

## RESULTS

### General characterization of SMs and SM cocktails for cell reprogramming

We first compiled a full list of SMs established thus far, based on a keyword meta-analysis of the literature. Comprehensive data mining with subsequent curation (see Methods) resulted in a total of 92 chemical compounds (Supplementary Table 1) that can either induce or enhance pluripotency, alone or in combination with TFs. These compounds for chemical reprogramming were named “Small Molecules” (SMs) because of their relatively low molecular weight [9], which ranges from 42.4 g/mol (LiCl) to 914.2 g/mol (Rapamycin). The vast majority of SMs represent organic compounds belonging to various chemical classes; however, among SMs were also several inorganic compounds (e.g., Lithium salts).

**Figure 1:**
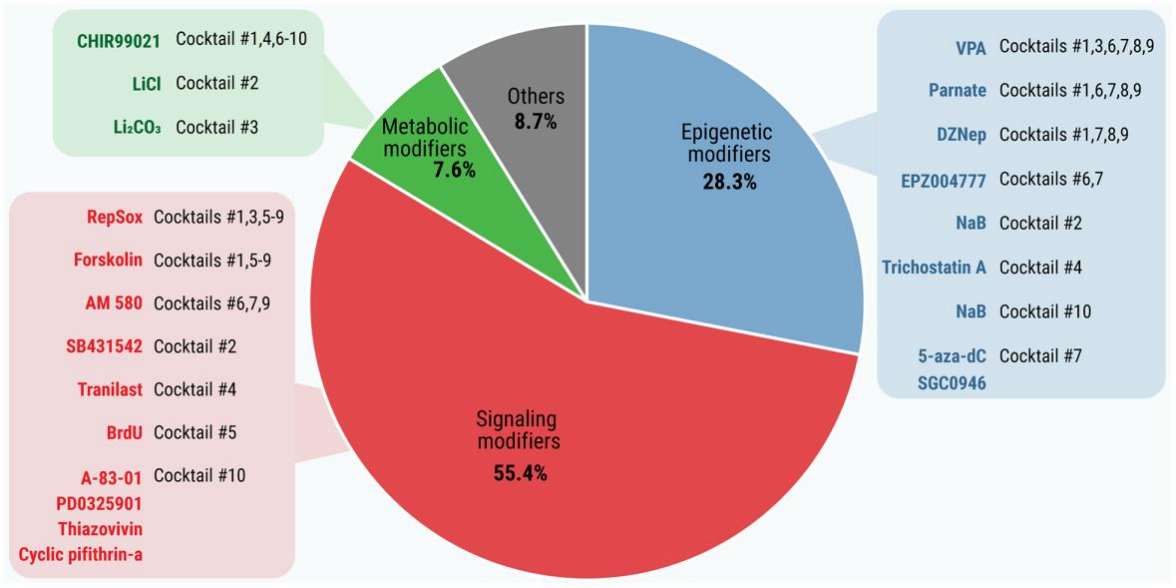
**Distribution of SMs by functional categories.** The basic biological activities of all SMs that induce or enhance pluripotency (n = 92) were extracted from the STITCH online tool, PubChem database and scientific literature. Functional categories of SMs were based on Gene Ontology Resource.

The analysis of the basic biological activities of the collected SMs revealed that they fall into three major categories (Figure 1 and Supplementary Tables 2–5): (i) signaling modifiers, (ii) epigenetic modifiers, and (iii) metabolic modifiers. It should also be mentioned that some SMs do not fall into definite categories or belong to more than one functional category.

The SMs with signaling activity represent the largest group (51 out of 92 compounds; 55.4%; Supplementary Table 2), followed by epigenetic (n = 26; 28.3%; Supplementary Table 3) and metabolic modifiers (n = 7; 7.6%; Supplementary Table 4). The most “popular” (i.e., most frequently used in SM cocktails) signaling modifiers include inhibitors of TGFβ and Hedgehog signaling, both involved in cell differentiation [15, 16]. In the epigenetic category, most SMs inhibit either methyltransferases (HMTs and DNMTs, 9 and 6, respectively) or HDACs (n = 4). Other molecules possess either dual activity (HDAC inducers and/or inhibitors, n = 3) or combined (inhibition of HMT+DNMT or DNMT+HDAC) activities. This, respectively, shifts the condensed form of chromatin (heterochromatin) towards a relaxed state (euchromatin) or decreases the level of DNA methylation, thereby ensuring more DNA to be available for transcription. Lastly, metabolic modifiers switch the metabolism from oxidative phosphorylation towards glycolysis, mostly through the inhibition of the GSK3 enzyme [17]. Other SMs (n = 8; 8.7%; Supplementary Table 5) include antioxidants, regulators of calcium transport, autophagy, etc.

To date, several combinations of SMs have been tested for cell reprogramming activity. Of them, 10 SM cocktails have been established. Their compositions, which vary from three [10] to ten [18] compounds, are presented in Supplementary Table 6. The common denominator for all these cocktails is that they are able to induce cell reprogramming, either full (pluripotent state) or partial (multipotent/progenitor cells), without transfection of stemness-related TFs.

A comparison between the cocktails revealed 22 non-redundant chemicals, presented in Table 1. It should be emphasized that each cocktail contains at least one SM from each of the epigenetic, signaling or metabolic activity categories, which coincide well with the results presented above. Of note, TGFβ inhibitors are presented in all cocktails. In particular, RepSox, which can replace Sox2 [19], is included in 7 of the 10 cocktails, and in the other three, the TGFβ inhibitors are replaced by SB431542 or Tranilast, both able to replace Sox2 [10, 19], or by A-83-01 [20]. Another frequently-used signaling modifier included Forskolin (found in six cocktails) or BrdU (in Cocktail *5*). The mentioned compounds can replace Oct4 [9, 21] (see Supplementary Table 1). The nuclear RARα selective agonist AM 580 and the synthetic retinoic acid receptor ligand TTNPB affecting the retinoic acid signaling pathway are used in four cocktails. As seen in Table 1, the GSK3 inhibitors (CHIR99021, LiCl or Li_2_CO_3_) which promote glycolysis are mandatory components of each reprogramming cocktail. Finally, all the cocktails include one or more epigenetic modifiers: HDAC inhibitors (VPA, NaB, Trichostatin A), DNMT inhibitors (5-aza-dC), the inhibitor of LSD1 acting on histone H3 (Parnate), and the inhibitors of histone methyltransferases (DZNep, EPZ004777, SGC0946). The common SMs are presented in the reprogramming cocktails in descending order: CHIR99021 = RepSox (n = 7), VPA = Forskolin (n = 6), Parnate (n = 5), DZNep (n = 4), AM 580 (n = 3), EPZ004777 (n = 2); other SMs are found only in one cocktail (see Table 1).

**Table 1:** Non-redundant SMs for reprogramming cocktails and their main bioactivities.

**SM**	**Main bioactivity**	**Cocktail**									
		**1**	**2**	**3**	**4**	**5**	**6**	**7**	**8**	**9**	**10**
CHIR99021	GSK3 inhibitor										
RepSox	TGFβ inhibitor [can replace Sox2]										
VPA	HDAC inhibitor										
Forskolin	cAMP activator [can replace Oct4]										
Parnate	Inhibitor of LSD1 acting on histone H3										
DZNep	Inhibitor of HMT EZH and SAH synthesis										
AM 580	Nuclear RARα selective agonist										
EPZ004777	DOT1L histone (H3K79) methyltransferase inhibitor										
NaB	HDAC inhibitor										
TTNPB	Synthetic retinoic acid receptor ligand										
BrdU	Synthetic analog of thymidine [can replace Oct4]										
LiCl	GSK3 inhibitor										
SB431542	TGFβ inhibitor [can replace RepSox]										
Tranilast	TGFβ inhibitor [can replace RepSox]										
Trichostatin A	HDAC inhibitor										
Li_2_CO_3_	GSK3 inhibitor										
5'-aza-dC	DNMT inhibitor										
SGC0946	DOT1L histone (H3K79) methyltransferase inhibitor										
Cyclic pifithrin-a	p53 inhibitor										
A-83-01	TGF-beta receptor inhibitor										
Thiazovivin	Rho Kinase (ROCK) inhibitor										
PD0325901	Potent MKK1 (MEK1) and MKK2 (MEK2) inhibitor										

### KEGG pathways enrichment analysis of SM targets

**Figure 2:**
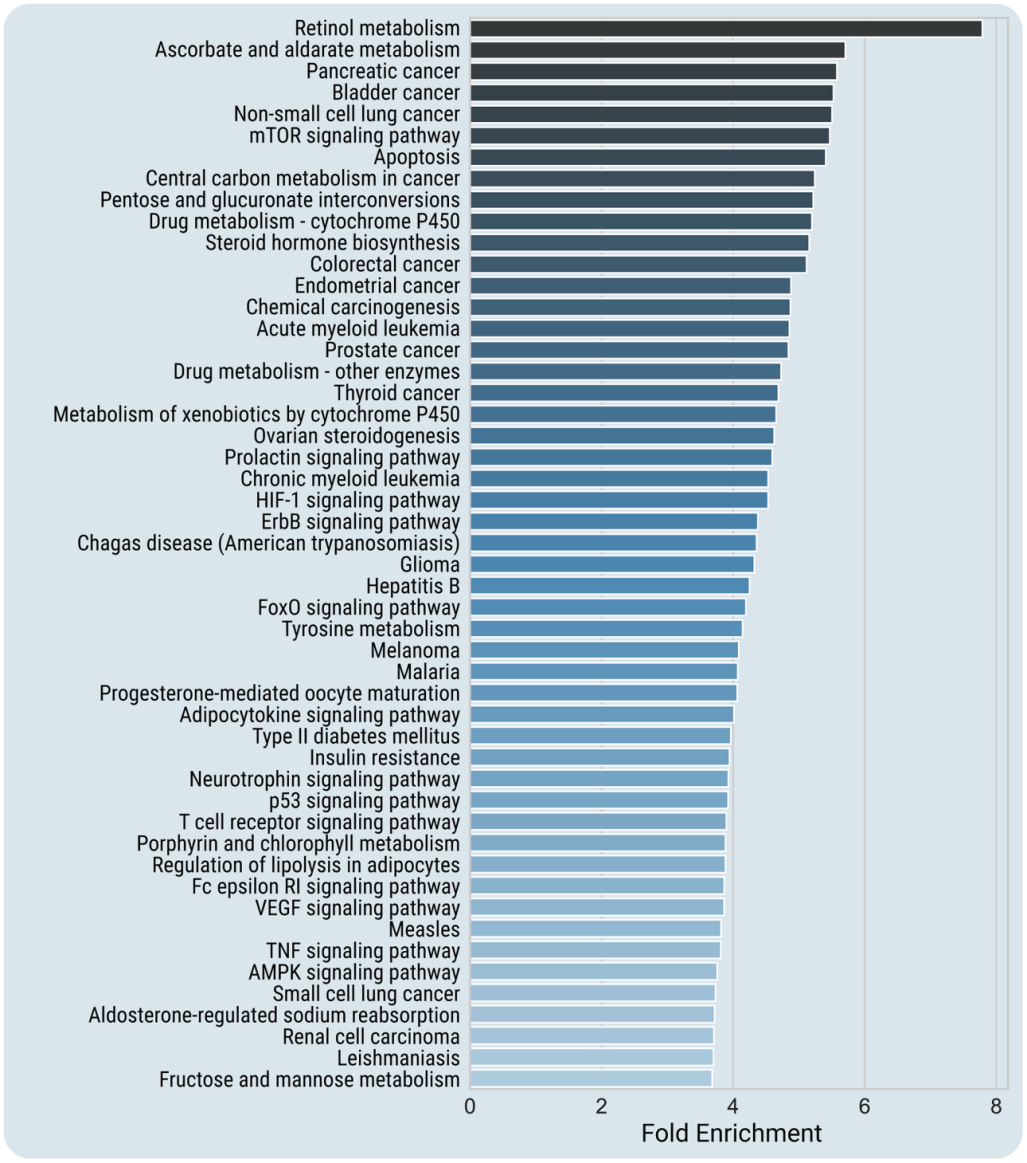
**Top enriched KEGG pathways of SM protein targets.** Enriched pathways at high confidence (p < 0.001 after Benjamini correction, with at least two-fold enrichment) are presented. Because of visualization limitations, only the top-most enriched 50 pathways are included in the figure. For a full list of the enriched pathways, see Supplementary Table 7, and for the enriched pathways for each SM cocktail, see Supplementary Table 8.

To get further insight into the mechanisms of chemically-induced reprogramming, we carried out an enrichment analysis for SM protein targets. For that purpose, we first used the STITCH database (https://pubmed.ncbi.nlm.nih.gov/26590256/) for extracting the chemical-protein interactions. Then, using the DAVID bioinformatics tools [22], we determined the enriched KEGG pathways of the found SM protein targets (in total, 1023). Figure 2 depicts the most enriched KEGG categories (p < 0.001 after Benjamini correction, with at least two-fold enrichment) among SM targets (for a full list of the enriched pathways, see Supplementary Table 7).

The most significantly enriched KEGG pathways include pathways associated with regulation of longevity such as mTOR signaling (p = 9.1E-18), AMPK signaling (p = 5.7E-17), Insulin signaling (p = 2.3E-13), FoxO signaling (p = 4.2E-23), and pathways involved in cell-cell and cell-extracellular matrix interactions (Focal adhesion, p = 3.7E-13, Adherens junction, p = 5.4E-06). Also, SM targets are over-presented in the signaling pathways associated with age-related diseases, including different types of cancer, type II diabetes mellitus (p = 7.7E-08), amyotrophic lateral sclerosis (p = 2.3E-04), and Alzheimer's disease (p = 2.5E-03). Among the enriched pathways are numerous growth-promoting pathways, cell survival (PI3K-Akt, p = 3.9E-19) or cell death (Apoptosis, p = 1.7E-18) signaling. Many enriched pathways are related to immune and inflammatory responses. Among them are the pathways related to innate immunity (Toll-like receptor signaling pathway, p = 1.8E-09; NK-cell mediated cytotoxicity, p = 2.6E-06), specific immune responses (T cell receptor signaling pathway, p = 5.3E-15; B cell receptor signaling pathway, p = 2.1E-07), and inflammatory signaling (Chemokine signaling pathway, p = 1.5E-13; Adipocytokine signaling pathway, p = 2.1E-11), etc. Not surprisingly, the enriched pathways include regulation of cell cycle (p = 3.5E-09), cell differentiation (Neurotrophins, p = 3.3E-18; TGFβ signaling, p = 9.4E-06), and Signaling pathways regulating pluripotency of stem cells (p = 2.8E-06).

### Network analysis of SM targets

**Figure 3:**
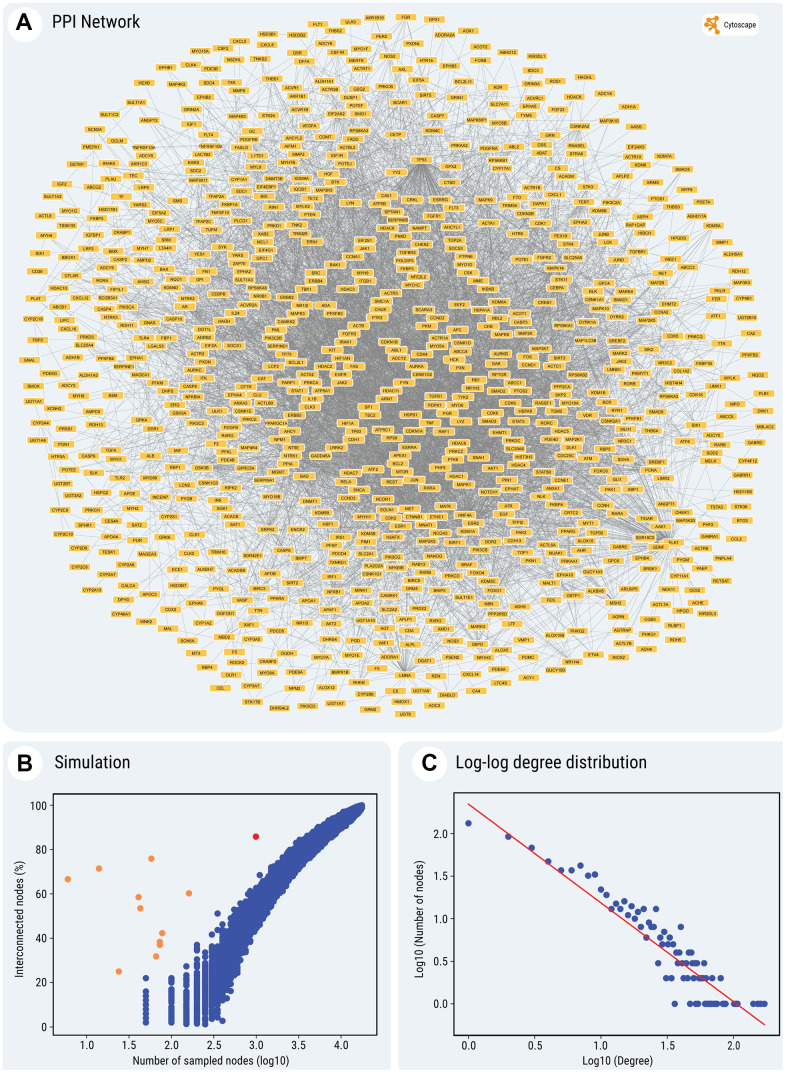
(**A**) Graphical output of the PPI network of the entire set of SMs' targets. (**B**) Simulation of expected interconnectivity given the size of a random sample. The observed interconnectivity of SMs' gene targets in the interactome, depicted by the red dot in the scatter plot and the observed interconnectivity of cocktails' gene targets, depicted by the orange dots, can be compared to the percentage of interconnected nodes (on the Y-axis), found in the largest continuous component of the network, for randomly sampled node sets. The plot shows the sampling of subsets of random interactome nodes, of various sizes (represented in a log10 scale on the X-axis, from 50 to 17,600 nodes). For each step, the interconnectivity was computed 100 times. Simulations were performed only for samples larger than 50 nodes, because of the increased variability of very small node sets. (**C**) The log-log plot of *P(k)* against *k*, illustrating scale-free topology of the network (for details, see the text and Methods). For all the nodes and edges in the network see Supplementary Table 9. (**A**, **C**) The construction and display of the network and the degree distribution regression were performed using Cytoscape, which pulls physical PPIs data determined *in vitro* and *in vivo* from the BioGRID database.

To further evaluate to what extent the SM targets interact between themselves, we determined their protein-protein interactions (PPIs), annotated in the BioGRID database [23]. These data are currently available for 991 out of 1023 SM target proteins. The analysis revealed that many of these targets interact with each other and exhibit multiple PPIs (in total, 6072 interactions). Remarkably, a significant fraction of the interacting SM targets (851 out of 991 proteins; 85.8%) forms a continuous network between themselves (Figure 3A). This fraction is significantly higher than expected by chance, i.e., higher than for the same number of randomly selected proteins with annotated PPIs (Figure 3B) (random sampling, mean ± SD: 52.8 ± 3.5%; z-score for observed value: 9.37).

Next, we aimed to understand the topology of the constructed network. To address this point, we calculated the distribution of node connectivity. The regression equation in Figure 3C (*P(k*) = 221 x *k* ^-1.16^) follows a power-law distribution of connectivity and indicates that the PPI network of SM targets has a scale-free topology, with an extremely high contribution of hubs to the average network connectivity.

**Figure 4:**
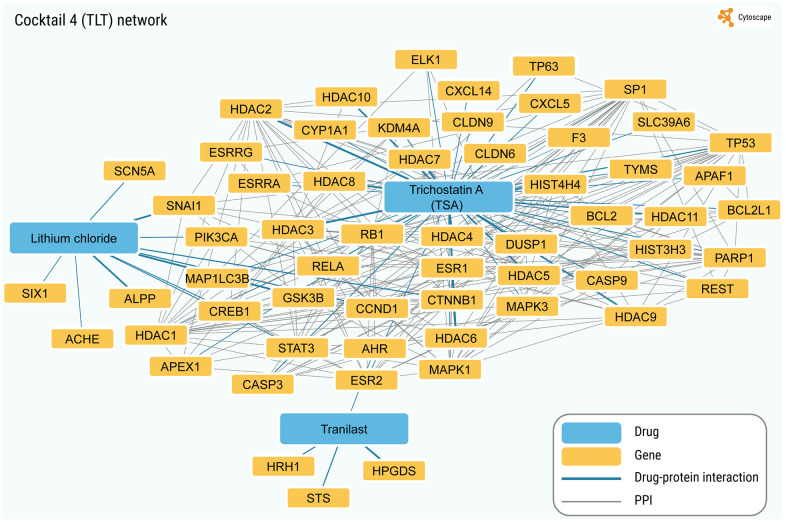
**The network with the highest interconnectivity (corresponding to the TLT cocktail).** In total, 58 protein targets are in the network. Continuous network without taking into account drug connectivity (chemical-protein interactions) includes 44 genes/proteins (75.9%; values for random sampling (mean ± SD): 4.5 ± 2.4; z-score for observed value: 30.03).

Using the same approach, we built the chemical-protein interaction and PPI networks for the ten SM cocktails used thus far for chemical reprogramming (see Supplementary Table 6). As seen in Figure 4 and Supplementary Figures 1–9, the total number of annotated protein targets in SM cocktails varied from 6 (Cocktail 10) to 174 (Cocktail 7), mostly falling around 50. In all cases, the fraction of proteins forming a continuous PPI network was extremely high (from 25% to 75.9%) for such small sizes of protein sets (Figure 3B), z-scores computed after random sampling being between 5.33 and 30. Collectively, the results obtained indicate that the SM targets are highly interconnected.

### Comparison of targets and pathways of SM cocktails with Yamanaka’s factors

**Figure 5:**
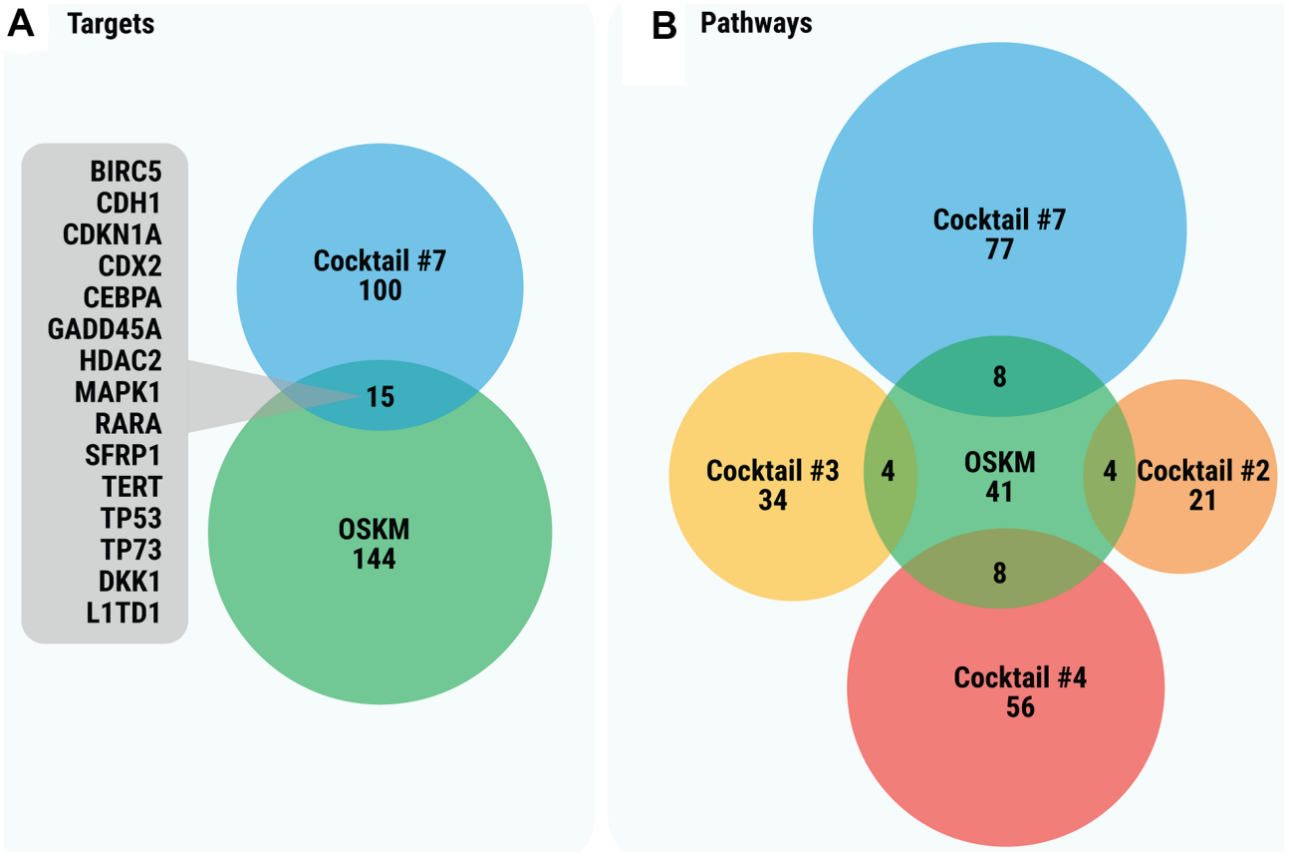
(**A**) Venn diagram of the gene targets of OSKM significantly overlapping with gene targets of cocktails. (**B**) Venn diagram of significantly overlapping enriched pathways for gene targets of SM cocktails and of OSKM. In order to simplify the figure, only statistically significant overlaps between OSKM and cocktails are displayed. Overlaps between pairs of cocktails are not shown.

It seems plausible that the cocktails for chemical cell reprogramming and TFs for iP, specifically Yamanaka’s factors (OSKM), have common targets (Figure 5A). However, their comparison showed that only the gene targets of Cocktail #7 (15 targets; p = 0.0033) overlap significantly with the targets of a “classical” combination of iP transcription factors (Figure 5A). Other cocktails overlap insignificantly (p > 0.05) with OSKM. Of note, Cocktail #7 has much more targets than any other cocktail for chemical reprogramming. In contrast to specific targets, several cocktails (#2, 3, 4 and 7) have significantly overlapped pathways with OSKM (Figure 5B). As seen in Table 2, most common pathways are cancer-related. Though not reaching the level of significance, the common pathways of other cocktails (#1, 5, 6, 8, 9 and 10) are also cancer-related.

**Table 2:** Overlapping pathways for targets of SM cocktails and OSKM.

**Pathways**	**Cocktails**									
	**#1**	**#2**	**#3**	**#4**	**#5**	**#6**	**#7**	**#8**	**#9**	**#10**
Pathways in cancer		*	*	*			*			
Chronic myeloid leukemia		*	*	*			*			
Prostate cancer		*		*			*			
Bladder cancer							*			
Small cell lung cancer				*						
Viral carcinogenesis			*	*			*			
HTLV-I infection		*		*			*			
Hepatitis B			*	*			*			
Epstein-Barr virus infection				*						
p53 signaling pathway							*			

Dark gray color with (*) depicts overlaps with p < 0.05. Light gray depicts the pathways with insignificant overlaps (p > 0.05).

### SMs as human metabolites

Most SMs are artificially synthesized chemicals. Of special interest is whether among the SMs are compounds that are natural (human) metabolites or their analogs. Overlapping the 92 SMs with the molecules found in the Human Metabolome Database - HMDB [24] gives a positive answer to this question: 28 compounds from the SM list are also found in HMDB (Table 3). The overlap is statistically extremely significant (p = 9.7E-83). For example, among SMs are essential natural metabolites (n = 8) including several vitamins (A, C, D), molecules belonging to fatty acids and their derivatives (NaB, PGE_2_), organooxygen (Fru-2,6-P2) and organonitrogen (Spermidine) compounds, and prenol lipids (Retinoic acid). Other “natural” SMs represent nutrients that integrate into the human body when consuming products of plant metabolism (n = 11). Interestingly, several of these compounds (e.g. EGCG, 7-hydroxyflavone, apigenin, curcumin, quercetin, resveratrol) are components of plant extracts that have been already shown to improve healthspan, in particular stress resistance and cognitive abilities [25]. Several SMs are medications, which under specific conditions can be found in the human body. Although they are not the products of human metabolism or essential nutrients, most of them are analogs of natural metabolites. For example, 5'-azaC or 5'-Aza-2'-deoxycytidine are analogs of the nucleoside cytidine; N-acetyl-cysteine is metabolized into L-cysteine, a precursor to the biologic antioxidant glutathione; Valproic acid (VPA) is a branched short-chain fatty acid derived from the naturally occurring Valeric acid [26].

**Table 3:** SMs as human metabolites.

**Name**	**Role in induced cell reprogramming** **(inducer/enhancer)**	**Chemical class**
5'-Azacytidine (5'-azaC)	Enhancer	Nucleotides and nucleotide derivatives
5'-Aza-2'-deoxycytidine	Enhancer	Nucleotides and nucleotide derivatives
7-hydroxyflavone	Enhancer	Flavonoids
90-D3 (Vitamin D3)	Enhancer	Steroids and steroid derivatives
Apigenin	Enhancer	Flavonoids
Caffeic acid	Putative enhancer or inducer	Cinnamic acids and derivatives
Chlorogenic acid	Putative enhancer or inducer	Fatty acids and derivatives
Curcumin	Enhancer	Diarylheptanoids
Dasatinib	Inducer	Benzene and derivatives
Dexamethasone	Enhancer	Steroids and steroid derivatives
EGCG	Enhancer	Flavonoids
Fisetin	Enhancer	Flavonoids
Forskolin	Inducer	Benzofurans
Fru-2,6-P2	Enhancer	Organooxygen compounds
Luteolin	Enhancer	Flavonoids
N-acetyl-cysteine	Enhancer	Amino acids and derivatives
Sodium Butyrate (NaB)	Inducer and enhancer	Fatty acids and derivatives
Prostaglandin E_2_	Enhancer	Fatty acids and derivatives
Quercetin	Enhancer	Flavonoids
Rapamycin	Enhancer	Macrolide lactams
Resveratrol	Enhancer	Stilbenes
Retinoic acid	Enhancer	Prenol lipids
SAHA	Enhancer	Benzene and derivatives
Spermidine	Enhancer	Organonitrogen compounds
Valproic acid	Inducer	Fatty acids and derivatives
Vitamin A (Retinol acetate)	Enhancer	Prenol lipids
Vitamin C (Ascorbic acid; Ascorbate)	Enhancer	Dihydrofurans
Zolpidem	Enhancer	Azoles

Furthermore, using STITCH tools [27], we found another 963 molecules that are similar (based on the STITCH drug similarity score) to the SMs that induce or enhance pluripotency, of them, 210 compounds (data not shown) are present in the Human Metabolome Database [24]. Among these compounds are neurotransmitters (serotonin, dopamine and GABA), fatty acids, and their derivatives involved in energy metabolism, such as citric acid, succinate and lactate. We determined the targets of these 210 chemicals, of the abovementioned eight human essential natural metabolites, and then compared them with the targets of all collected SMs (n = 1,023) and SM cocktails (n = 204) (Supplementary Table 10). As seen in the Supplementary Table 10, there is an extremely significant (p < E-25, Fisher test) overlap between the targets of the 210 SM-like chemicals (n = 4,614) and the targets of all SMs or the targets of SM cocktails. The common targets cover more than 76% (782 of 1023 targets) and 65% (132 out of 204 targets), respectively. Also, an extremely significant overlap was found for the targets of the abovementioned 8 human natural metabolites (n = 318) and the targets of SM cocktails (21%, 43 of 204).

## DISCUSSION

Until now, the selection of SMs for chemically-induced pluripotency or cell reprogramming was done mainly on an empirical basis, and no analysis of SMs and their targets has been undertaken. Several reviews published in the past [28–32] focused on specific aspects of SMs but none of them provided a “systemic” view. Our comprehensive data mining with subsequent data curation revealed 92 SMs that have been reported in connection to cell reprogramming. Most of the SMs were primarily used as enhancers of iP, i.e., for increasing the efficiency of cocktails containing TFs (e.g., Yamanaka’s factors) [30, 33, 34]. Of note, to a lesser degree, SMs were also used as enhancers of cell reprogramming in SM cocktails without TFs. Apart from cell dedifferentiation, in the last years, SMs have also been used for cell transdifferentiation (for a review see Xie et al., 2017 [13]). Still, we found among the studied SMs many that could be classified as stand-alone inducers of cell reprogramming. These SMs were able to induce cellular reprogramming by themselves, thus either fully replacing the essential TFs [9, 10] or by increasing their expression [35, 36]. For example, Forskolin can replace Oct4, while RepSox can substitute Sox2 (see Supplementary Table 1). Besides the classical iP by means of the combinations of overexpressed TFs (e.g., Yamanaka’s factors, OSKM), a total of ten cocktails that contain SMs only with cell reprogramming activity have been established and tested thus far.

Functional analysis of SMs and their targets revealed that they are distributed between three major categories: epigenetics, intra- and inter-cellular signaling, and metabolic “switchers”. All these categories appear to be mandatorily presented in each SM cocktail to induce cell reprogramming. Specifically, it seems that sufficient components for a “minimal reprogramming” cocktail have to include an inhibitor of HDAC (e.g. VPA or NaB), an inhibitor of TGFβ signaling (e.g. RepSox), and GSK3-inhibiting SMs (e.g. CHIR99021 or LiCl). This assumption was further confirmed by the KEGG pathways enrichment analysis. The unusually significant enrichment of epigenetic and signaling pathways highlights their importance in chemical iP. Remarkably, many enriched pathways were related to aging, longevity and age-related diseases, thus presumably connecting them with the processes of cell reprogramming. This notion has recently been supported experimentally by demonstrating induction of cellular senescence by activation of OSKM, *in vitro* [37] and also *in vivo* on i4F reprogrammable mice [38–40]. Yet, this does not minimize the potential importance of pathways that are only slightly enriched or are not enriched at all. For example, Glycolysis/Gluconeogenesis pathway appears in our analysis as a marginally significantly enriched pathway (p = 0.051), although it is a well-recognized metabolic pathway for cell reprogramming; moreover, it is well known that the pluripotent stem cells rely on glycolysis rather than OXPHOS (reviewed by [3]). The possible explanation for this result is most likely related to the small number of glycolytic enzymes among the SM targets, relative to the total number of targets. Further strengthening the importance of metabolic components of iP is the observation that the HIF-1 signaling pathway is among the most significantly enriched pathways (fold enrichment = 5, p < 2.0E-20). Indeed, the hypoxia-inducible factor 1 alpha (Hif1alpha) activates glycolysis and concomitantly promotes telomerase expression and enhances self-renewal of stem cells [41]. Another important observation is that the main transcription factors of pluripotency, Oct4 and Nanog, can directly induce expression of the key glycolytic enzymes hexokinase 2 and pyruvate kinase M2, thus delaying differentiation and preserving pluripotency of ESCs [42]. In turn, the genes involved in the control of glucose uptake (GLUT3) and metabolism (PKM2) are also involved in the regulation of Oct4 expression [43]. For unclear reasons, some promising SMs have not been used in reprogramming cocktails developed thus far. For example, vitamin C (see Table 3 and Supplementary Tables 1, 5) was shown to modulate the TET enzymes, which promotes demethylation of histones and DNA, with subsequent enhancing cell reprogramming induced by OSKM [44–46], however it was not yet evaluated in combination with any SM cocktail.

It is still a matter of debate whether SMs act independently of each other in triggering cell reprogramming, or if they act in a cooperative, epistatic manner. The latter suggests the interactions between their targets, including direct (physical) interactions. With this in mind, we analyzed the connectivity and interconnectivity of targets of SMs and SM cocktails. The network analysis indicates that their targets are highly interconnected and form PPI networks with a scale-free topology that confers robustness and persistent connectivity. This means that: (i) the SM targets probably act in a cooperative manner to induce cell reprogramming; (ii) a scale-free topology of SM targets ensures higher integrity of the network and its resistance to random attacks [47, 48], thus making the cell reprogramming process highly reliable.

Recently, we hypothesized that cell reprogramming is a natural process that is triggered and regulated via two major networks – a genetic one (triggered by transcription factors, e.g. OSKM) and a chemical one (controlled by metabolites, e.g. similar to SMs) [3, 49]. In line with this hypothesis are our data demonstrating that: (i) a large number of SMs (28 of 92; Table 3) used for cell reprogramming are found in the human metabolome (derivatives of nucleotides, fatty acids, etc.), and (ii) many more metabolites (over 200) are functionally similar to SMs, thus offering the potential of being cell reprogramming agents. In addition to the chemical factors, environmental factors such as hypoxia and/or hypercapnia (which eventually act as chemical factors, namely through low concentrations of oxygen and high concentrations of carbon dioxide) may greatly influence the cell dedifferentiation process [3, 50]. It should be mentioned again (see above) that hypoxic/hypercapnic microenvironment associated with a low reactive oxygen species (ROS) generation and activation of glycolysis, is essential for maintenance and proper functioning of dedifferentiated cells.

Further supporting our hypothesis are the data on the common targets of SM cocktails and Yamanaka’s factors. This comparison revealed an insignificant overlap between the SM cocktails’ targets and OSKM, except for Cocktail #7. The lack of common targets between the cocktails and Yamanaka factors was quite a surprising observation. More prominent overlap was however observed between pathways, meaning that despite different targets, both SM cocktails and Yamanaka’s factors “use” more or less the same pathways.

Altogether, this suggests that the two systems, chemical (SMs) and genetic (TFs), might cooperate to increase the efficiency of cell reprogramming. Interestingly, the overlapping pathways for SM cocktails and OSKM targets are mainly cancer- or virus-related but not related to key reprogramming processes, such as demethylation and chromatin decondensation or pluripotency pathways, as it might be expected. One of the reasons could be rooted in statistical issues. In Table 2, only the pathways significantly overlapping with at least one SM cocktail, are presented. Another important point is that cancer-related pathways are not “purely" cancer pathways, but include many components related to cell division and reprogramming. For example, Wnt/β-catenin and MAPK signaling pathways are known for their role in cell dedifferentiation [51, 52]. These pathways are also well known for their involvement in carcinogenesis [53].

Although beyond the scope of the present study, it is worth mentioning that there is a significant overlap between the collected 92 SMs and the compounds found in the DrugAge database [54] (n = 20 drugs; p = 4.95E-15). Among the common drugs are Rapamycin, Valproic acid, Caffeic acid, and Lithium chloride. Similarly, there is a large overlap between the SM targets and the longevity-associated genes (LAGs) hosted in the GenAge database [55] (n = 132, p = 3E-88 for human LAGs and n = 136, p = 5E-24 for human orthologs of model organism LAGs). Lastly, SM targets also overlap with the list of genes related to cellular senescence (CS) from the CellAge database [2] (n = 85, p = 1E-42). As a point for further investigation is testing the established or newly constructed SM cocktails *in vivo*. In this regard, testing SM cocktails in the naked mole-rat model could be of particular interest as induction of pluripotency in the cells of this animal requires special conditions and is not always achievable [56–58].

All in all, SMs and their relationship with TFs definitely warrants further investigation which could probably shed more light on the mechanisms of cell reprogramming and will be helpful for developing the most optimal SM cocktails with effects on CS, aging and longevity.

## MATERIALS AND METHODS

### Data sources

Data on SMs for cell reprogramming were gathered from publicly available literature, using PubMed NCBI (http://www.ncbi.nlm.nih.gov/pubmed/) and Google Scholar (https://scholar.google.com/). Additional data about the chemical and biological properties of SMs were obtained from PubChem [59], https://pubchem.ncbi.nlm.nih.gov/ and from the Human Metabolome Database (HMDB) [24], http://www.hmdb.ca/. Briefly, HMDB contains the collection of small molecules found in the human body, including nucleic acids, carbohydrates, lipids, peptides, amino acids, organic acids, biogenic amines, vitamins, minerals, food additives, drugs, cosmetics, contaminants, pollutants, and other chemicals that enter the human body [24].

### Data mining and organization

The papers were searched using the following keywords: “induced pluripotency”, “chemically induced pluripotency”, “chemical reprogramming”, “chemically induced dedifferentiation”, “induction of pluripotency by small molecules”. In order to be included in the analysis, each article had to contain data: (i) on SM(s) or their cocktail(s) that either induced or enhanced cellular reprogramming; (ii) on the bioactivity of the SMs; and (iii) on the SM dosage and cell type. According to their role in cell reprogramming, the compounds found were divided into two major groups of molecules: iP inducers and iP enhancers. Since it was not always possible to definitely link the compounds to one of the groups, as in some cases a given compound was considered an inducer and in other cases an enhancer, these entities were marked as “inducer and/or enhancer”. From each paper the following data were collected and manually curated: (i) the name(s) of SM(s) that either induce or enhance pluripotency, with or without TFs; (ii) the effect of SM(s) on the iP efficiency; and (iii) whether a given SM can substitute the pluripotency-associated TFs. The collected SMs were organized in a table as shown in Supplementary Tables 1, 6. The data regarding each compound included: common name, formula, molecular weight (MW), main bioactivity/target(s), comments relevant to cellular reprogramming, link to PubChem references, PMID. Only the SM cocktails which induced cell reprogramming (not necessary to the stage of iPSCs) without TFs were included in the analysis.

### Drug-protein interaction network

To determine the protein targets of the collected SMs, we used the STITCH database (version 5.0), http://stitch.embl.de/, one of the largest repositories of chemical-protein interactions [27], which include direct (physical) and indirect (functional) interactions. For the scope of the analyses in this study, text-mining and predicted interactions were excluded. If not indicated otherwise, a confidence score of medium stringency (0.4) was used for including interaction in the analysis. Drug similarity analysis was performed using the STITCH tool as described by Kuhn et al. [60].

### Gene targets overlap

To obtain the list of OSKM transcription factors the TRRUST database [61], https://www.grnpedia.org/trrust/, was used. The overlaps between gene targets of drug cocktails and OSKM transcription factors were calculated using only the genes that are present in both STITCH and TRRUST databases. In order to compute the overlap between gene targets of SMs and GenAge [55], https://genomics.senescence.info/genes/index.html, two lists of longevity-associated genes (LAGs) were used: i) the manually curated list of human LAGs from GenAge, build 20 and ii) the human orthologs of model organisms LAGs from GenAge, build 20. Orthologs of genes were computed using a script developed in our lab, that queries the database InParanoid 8 [62], https://inparanoid.sbc.su.se/cgi-bin/index.cgi. For stringency, we selected for each gene only inparalogs with scores of 1.0. The significance of the overlaps with GenAge [55] and CellAge [2] - https://genomics.senescence.info/cells/, was computed using Fisher’s exact test.

### SMs overlap with chemical databases

The overlaps between: i) the list of SMs and HMDB, and ii) the list of SMs and DrugAge [54] were calculated using the PubChem IDs of the compounds as identifiers. The significance of the overlap was computed using Fisher’s exact test and considering all PubChem and all DrugBank compounds, respectively, as background.

### KEGG pathways and gene ontology enrichment analysis

Functional and pathway enrichment analyses were performed with the DAVID Bioinformatics Resources tool, version 6.8 [22], https://david.ncifcrf.gov. Statistical significance of enrichment was evaluated using default parameters set in DAVID. A threshold of 0.001 was used for the adjusted *P*-value.

### Protein-protein interaction networks

Protein-protein interaction (PPI) data were taken from the BioGRID database [23], http://thebiogrid.org, human interactome, Build 3.5.177. The PPI network construction and analyses were performed using Cytoscape [63], http://www.cytoscape.org, version 3.7.1. Prior to any network analyses, genetic interactions, self-loops, duplicate edges and interactions with proteins from other species were removed from the interactome, and the remaining network was used as a control. The interconnectivity was computed as the fraction of nodes in the largest connected component out of the input gene set, by using the breadth-first search algorithm. Modeling the relationship between node subset size and interconnectivity in the human interactome was carried out by randomly sampling subsets of nodes in the interactome, with a sample size varying from 50 to 17,600 nodes (step of 50). In this case, sampling was performed 100 times for each subset size. In order to evaluate the statistical significance of the observed network interconnectivity for cocktails and SMs gene targets, random sampling from the BioGRID network was performed 1000 times, for a subset of nodes of equal size to each evaluated network. For each set of random samplings, average interconnectivity, standard deviation and z-score of the observed interconnectivity were computed.

For a joint protein-drug network, the protein targets of the collected SMs, determined from the STITCH database, were used together with PPIs from BioGRID.

## Abbreviations for SM cocktails

### Cocktail 1 (VC6TF + TTNPB + DZNep)

Valproic acid (V, VPA)

CHIR99021 (C)

RepSox (6)

Parnate (T)

Forskolin (F)

DZNep

TTNPB

### Cocktail 2 (NLS)

NaB

LiCl

SB431542

### Cocktail 3 (VCR)

Valproic acid

CHIR99021

RepSox

### Cocktail 4 (TLT)

Trichostatin A (TSA)

Li2CO3

Tranilast

### Cocktail 5 (BrdUC6F)

BrdU

CHIR99021

RepSox

Forskolin

### Cocktail 6 (VC6TF + AM 580 + EPZ004777)

VPA

CHIR99021

RepSox

Parnate

Forskolin

AM 580

EPZ004777

### Cocktail 7 (VC6TF + AM580 + DZNep + 5-aza-dC + SGC0946 + EPZ004777) SMs and their protein targets.

VPA

CHIR99021

RepSox

Parnate

Forskolin

AM 580

DZNep

5-aza-dC

SGC0946

EPZ004777

### Cocktail 8 (VC6TF + DZNep)

VPA

CHIR99021

RepSOX

Parnate

Forskolin

DZNep

### Cocktail 9 (VC6TF + AM 580 + DZNep)

VPA

CHIR99021

RepSox

Parnate

Forskolin

AM 580

DZNep

### Cocktail 10 (CNɑATP)

CHIR99021

NaB

cyclic pifithrin-a (ɑ)

A-83-01

Thiazovivin

PD0325901

## Supplementary Material

Supplementary Figures

Supplementary Table 1

Supplementary Table 2

Supplementary Table 3

Supplementary Table 4

Supplementary Table 5

Supplementary Table 6

Supplementary Table 7

Supplementary Table 8

Supplementary Table 9

Supplementary Table 10
