# Supplementary material for: Small molecules for cell reprogramming: a systems biology analysis
Source: Aging (Albany NY). 2021 Dec 17;13(24):25739–62. doi: 10.18632/aging.203791 (PMC8751603; doi:10.18632/aging.203791)
Supplement: Supplementary Figures [file aging-13-203791-s001.pdf]

SUPPLEMENTARY FIGURES

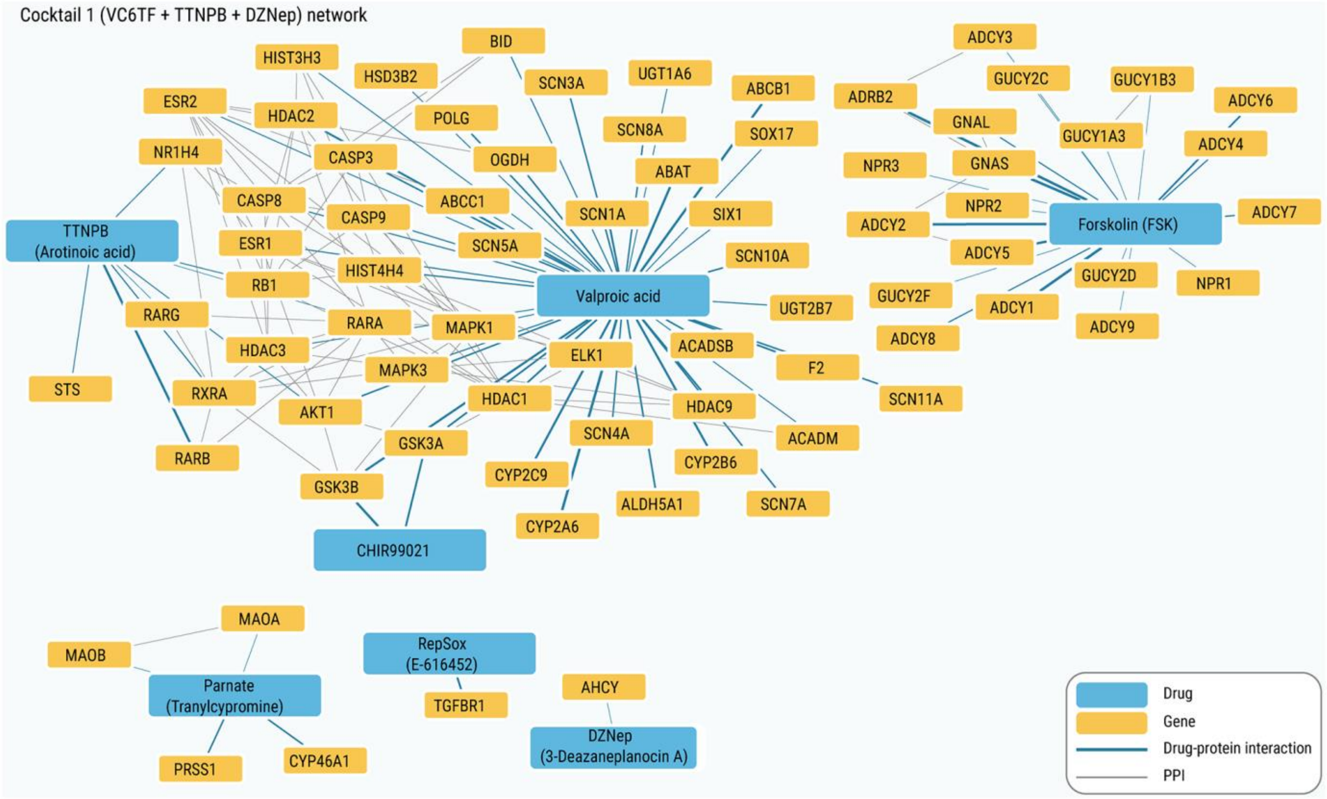

Supplementary Figure 1. The network of cocktail 1 (VC6TF + TTNPB + DZNep) SMs and their protein targets.

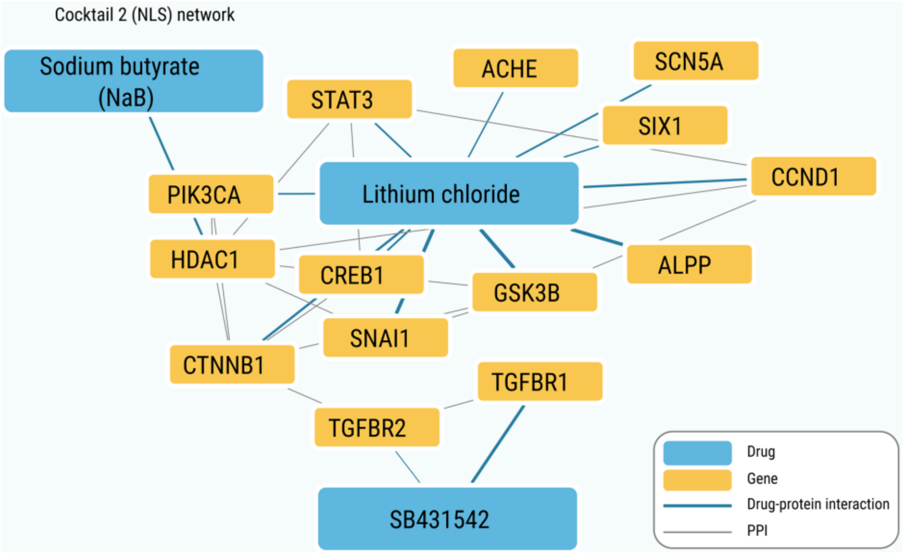

Supplementary Figure 2. The network of cocktail 2 (NLS) SMs and their protein targets.

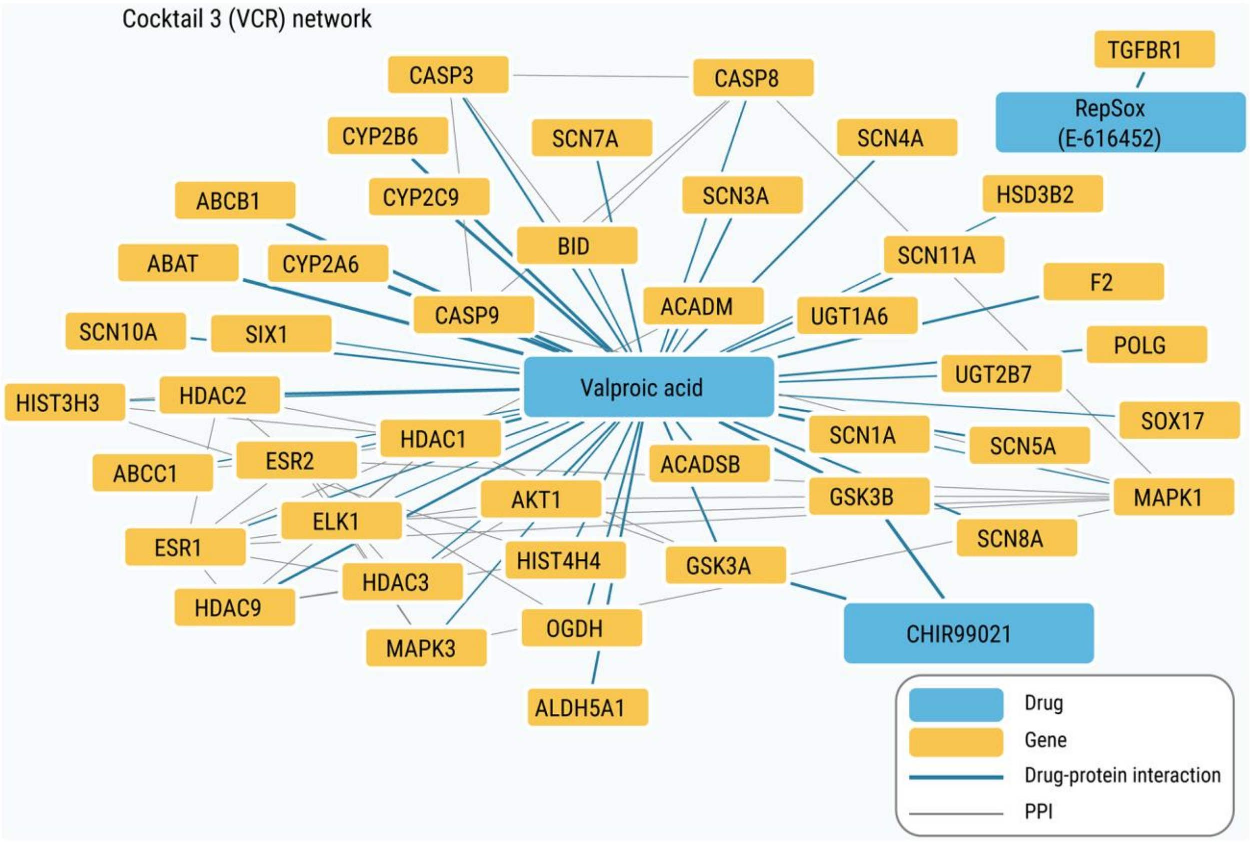

Supplementary Figure 3. The network of cocktail 3 (VCR) SMs and their protein targets.

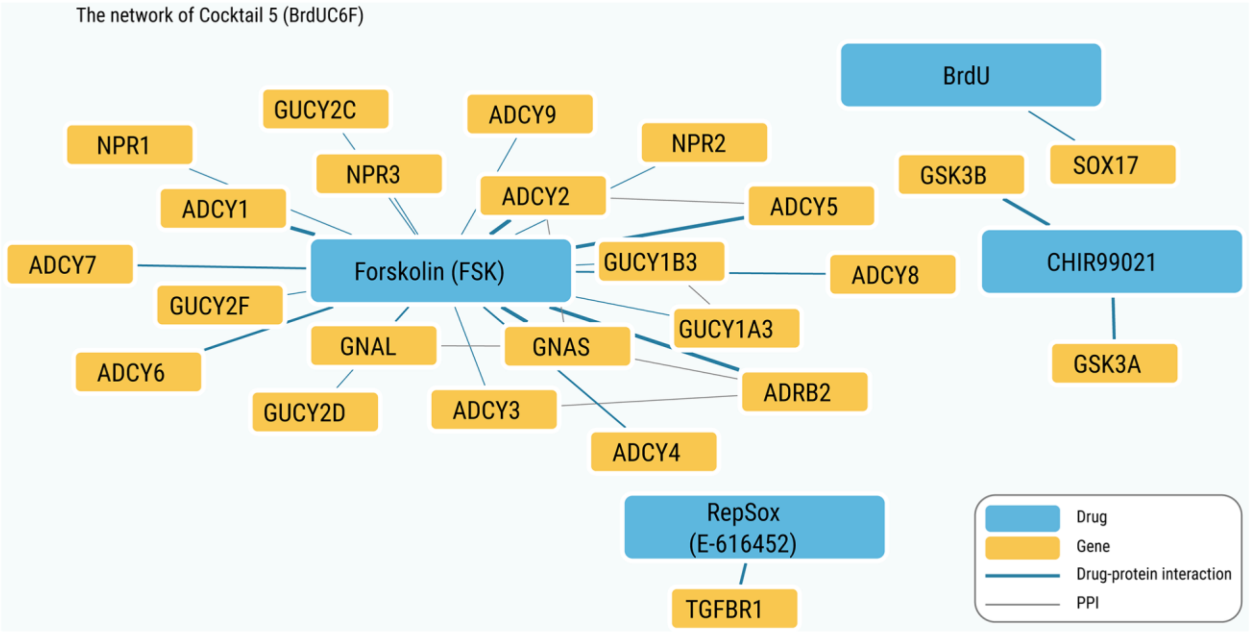

Supplementary Figure 4. The network of cocktail 5 (BrdUC6F) SMs and their protein targets.

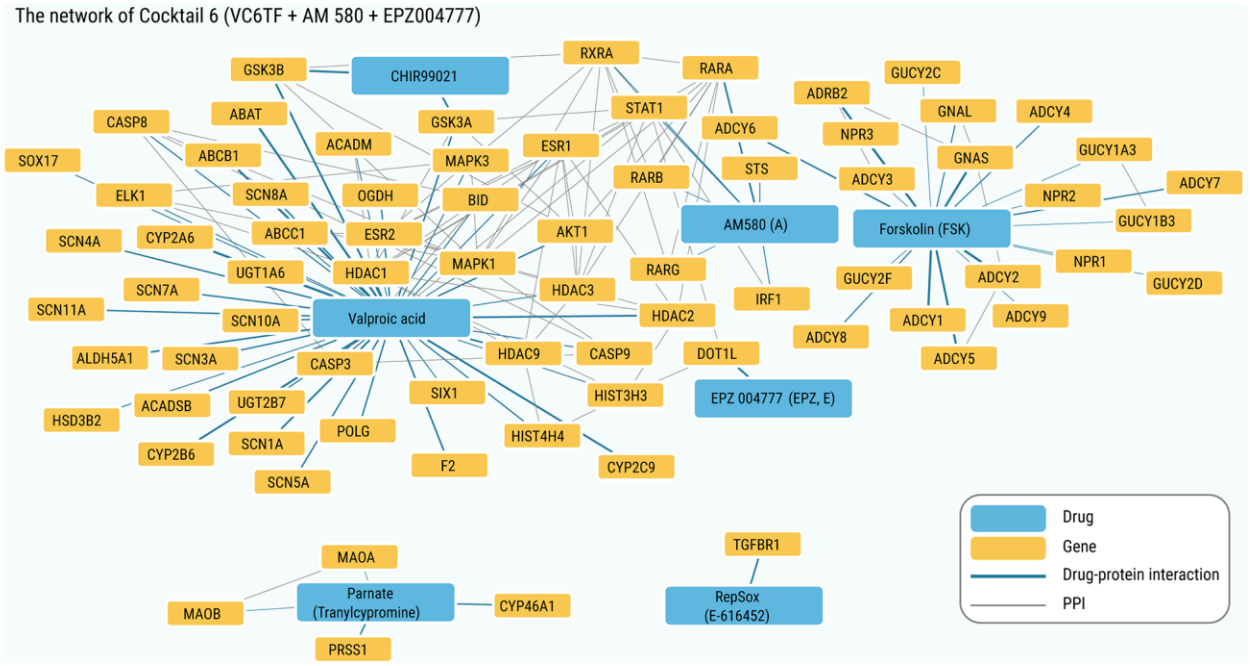

Supplementary Figure 5. The network of cocktail 6 (VC6TF + AM 580 + EPZ004777) SMs and their protein targets.

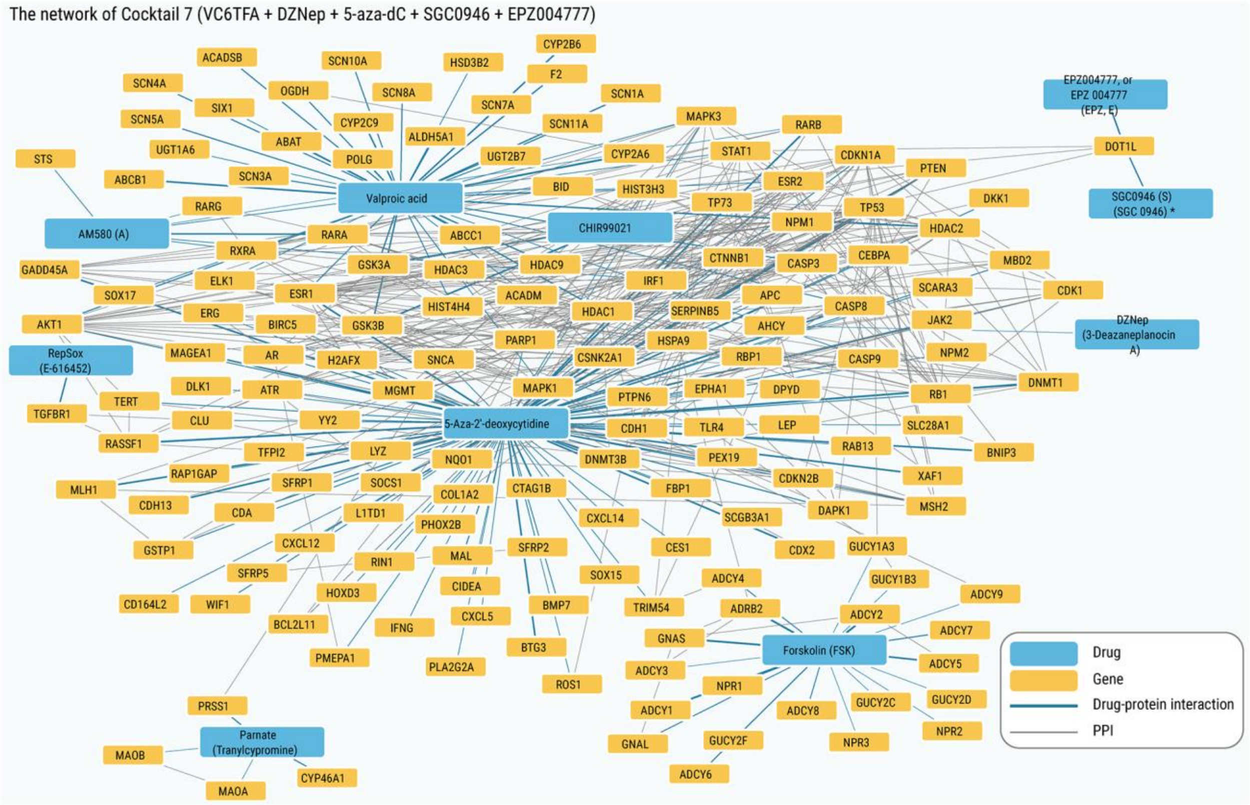

Supplementary Figure 6. The network of cocktail 7 (VC6TFA + DZNep + 5-aza-dC + SGC0946 + EPZ004777) SMs and their protein targets.

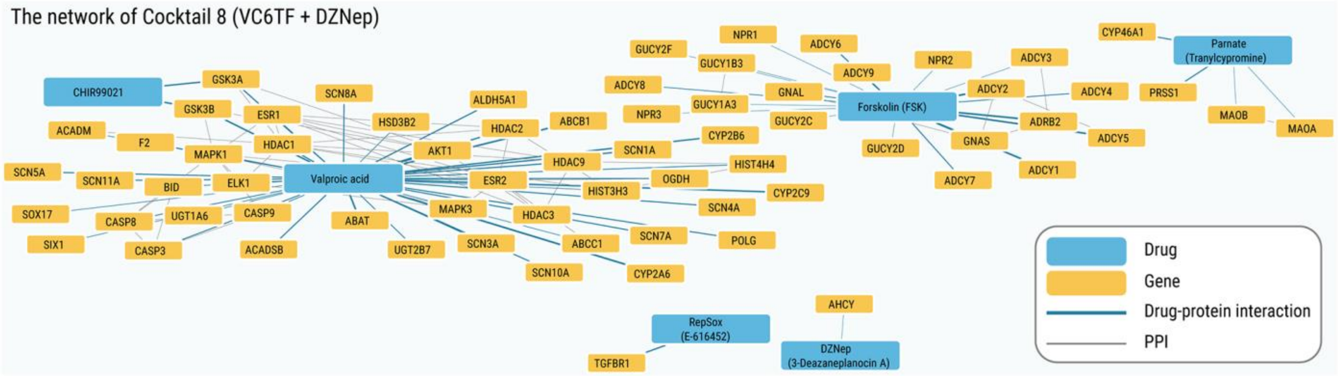

Supplementary Figure 7. The network of cocktail 8 (VC6TF + DZNep) SMs and their protein targets.

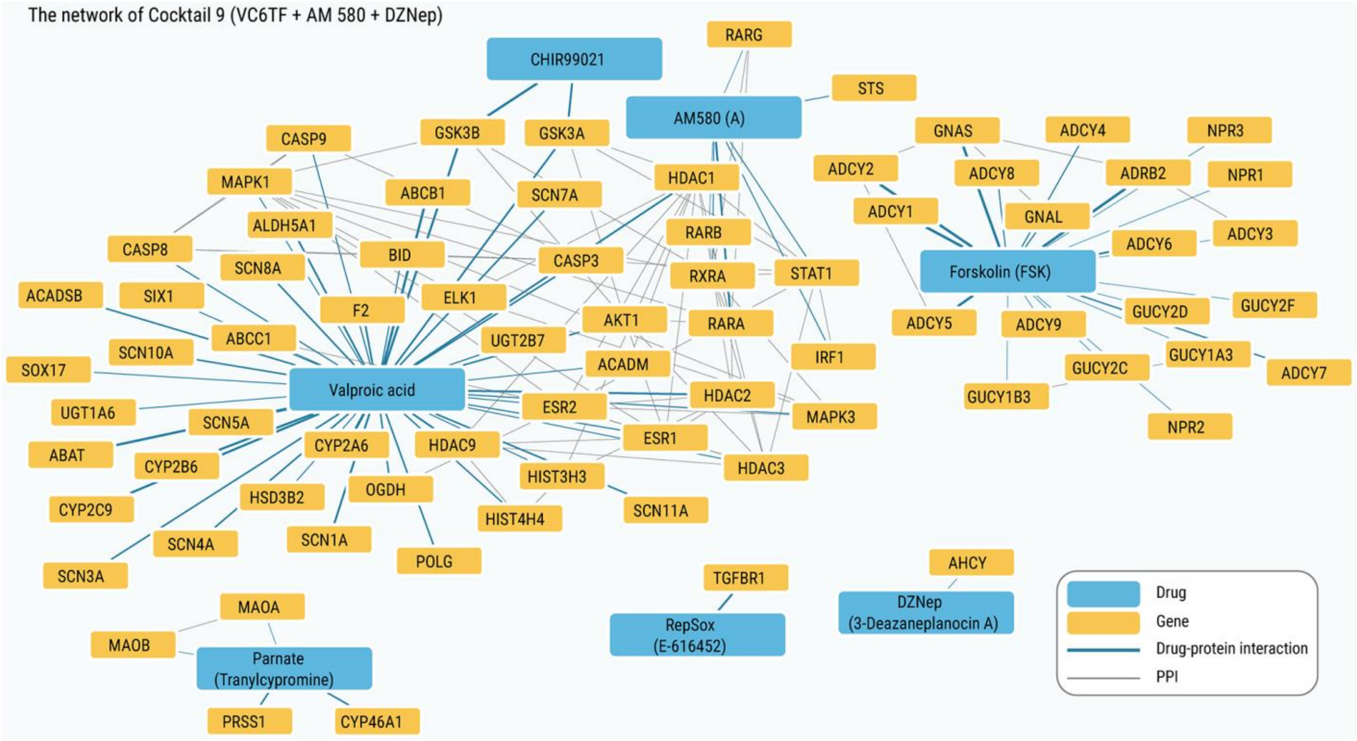

Supplementary Figure 8. The network of cocktail 9 (VC6TF + AM 580 + DZNep) SMs and their protein targets.

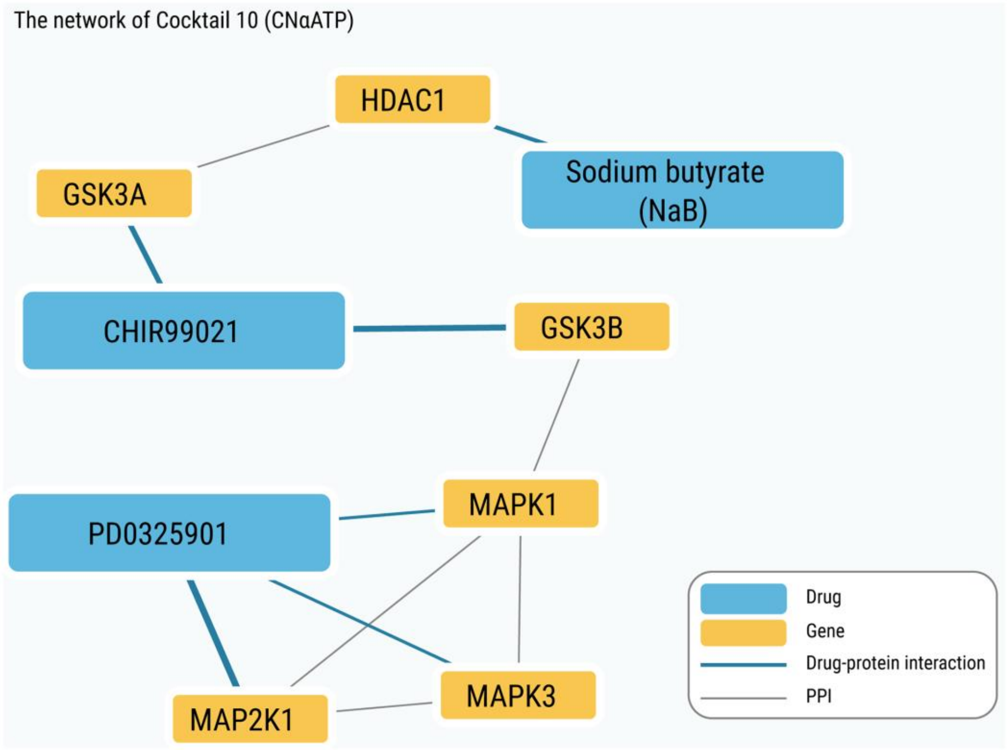

Supplementary Figure 9. The network of cocktail 10 (CNαATP) SMs and their protein targets.
